# Role of Imaging in the Management of High-Risk Endometrial Cancer

**DOI:** 10.7759/cureus.19286

**Published:** 2021-11-05

**Authors:** Catarina Silva, Carolina Carneiro, Teresa Margarida Cunha

**Affiliations:** 1 Radiology, Hospital Pedro Hispano, Matosinhos, PRT; 2 Radiology, Hospital de Braga, Braga, PRT; 3 Radiology, Instituto Português de Oncologia de Lisboa Francisco Gentil, Lisboa, PRT

**Keywords:** pet/ct, radiology, mri, gynecological cancer, endometrial cancer

## Abstract

Endometrial cancer (EC) is the second most frequent gynecological malignancy worldwide with an overall favorable prognosis. However, there is a subgroup of patients with a higher rate of recurrence and worse prognosis who benefit from a specific pre- and post-treatment radiological evaluation that allows the adjustment of the therapeutic attitude towards the biology of the tumor.

The main factors that determine high-risk disease are non-endometrioid tumor histology, histopathological grade 3, lymphovascular space invasion (LVSI), myometrial invasion ≥50%, and cervical stroma involvement. Radiological evaluation helps identify high-risk cases prior to surgical staging and is an important tool both in pre-treatment and in case of clinical recurrence suspicion.

As for imaging techniques, both transvaginal ultrasound and MRI can assess local tumor extent while CT and positron emission tomography/CT (PET/CT) help assess lymph nodes and distant metastases.

The central purpose of this article is to review the specific factors that determine high-risk endometrial cancer, and the main specificities in the pre-treatment and follow-up evaluation according to the most recent international guidelines.

## Introduction and background

Endometrial cancer (EC) is an increasingly challenging gynecological cancer, being the most common gynecological malignancy in developed countries [1,2]. It is more likely to occur in postmenopausal women, although 20% to 25% are diagnosed before menopause [3,3]. Its incidence is increasing worldwide and the risk of EC is positively correlated with obesity and conditions associated with metabolic syndrome [1,4]. In addition, conditions involving excess estrogen, including long-term use of unopposed estrogen, early menarche, and late menopause, predispose women to EC. Tamoxifen, which has pro-estrogenic effects in the uterus, approximately doubles the risk of endometrioid and non-endometrioid types of EC. Parity and oral contraceptive are factors that protect against EC [2,5,6].

Despite its relatively high prevalence, the overall prognosis of EC is favorable with a worldwide low mortality rate estimated at 2.4% [2,3,7]. Approximately 82% of EC are diagnosed at an early stage of the disease, with abnormal vaginal bleeding or discharge being the initial symptom, which contributes to its global good prognosis [4].

Biologically and clinicopathologically, EC is subdivided into two types [1,5]. The type I or estrogen-dependent carcinomas (>80%) are composed of grade 1 and 2 endometrioid carcinoma. It is the most common type of EC that may arise from complex atypical hyperplasia and is associated with prolonged unopposed estrogen exposure. The diagnosis of Type I tumors is usually made at earlier stages which confers a relatively good prognosis [6,8].

The type II or estrogen-independent carcinomas account for 10% to 20% of EC and are composed of grade 3 endometrioid carcinoma and all non-endometrioid tumors. This type of tumor is less hormone-sensitive and generally more aggressive with a poorer prognosis than type I [6,7-9]. 

Although EC is surgically staged, it is important to identify the extent of the disease before surgery to optimize the best modality approach and treatment [7,8,10]. Preoperative imaging is helpful in patients with suspected extrauterine disease and allows the detection of locoregional advanced disease, identification of suspicious lymph nodes, and distant metastasis [7,11]. Cross-sectional imaging techniques are complementary modalities for surgical evaluation of EC [12] and play an important role both in the pretreatment and follow-up assessment of the disease [1,7].

The central purpose of this manuscript is to review the role of imaging in high-risk endometrial cancer, particularly in diagnostic and staging assessment, and post-treatment surveillance of these patients. 

## Review

Endometrial cancer stages

Surgical staging of EC replaced clinical staging by the International Federation of Gynaecology and Obstetrics (FIGO) Committee on Gynecologic Oncology in 1988, and greater knowledge of the tumor biology allowed an update in the FIGO staging system in 2009 [8,12-14]. The complete surgical staging procedure includes hysterectomy with bilateral salpingo-oophorectomy, with biopsies of eventual suspicious peritoneal lesions and cytology of peritoneal washings as well as pelvic and retroperitoneal lymphadenectomy [8,15]. 

The FIGO stage IA and IB are divided accordingly by the depth of myometrial invasion (less or ≥50 % myometrial invasion, respectively). Endometrial cancer is classified as stage II if there is cervical stromal invasion, which represents a higher risk of lymphovascular space invasion and is consequently associated with a poorer prognosis. For assigning stage III, local tumor spread must be beyond the uterus but still present within the female pelvis. Subset IIIA represents the invasion of serosa or adnexa involvement while subset IIIB shows direct parametrium or vaginal infiltration. Stage IIIC indicates nodal involvement and is subdivided into pelvic (stage IIIC1) and para-aortic (stage IIIC2) nodal involvement. Stage IV disease represents the direct full-thickness invasion of the bladder or rectal mucosa (stage IVA) or the presence of distant metastases (stage IVB) [6-9,13].

Prognostic tumor characteristics for high-risk disease

Surgical staging can not be replaced by imaging. However, preoperative risk stratification remains an important tool to predict patient prognosis [4,7]. Furthermore, the selection of preoperative high-risk patients helps define the surgical approach and the need and extent of lymph node sampling or extended lymphadenectomy [7,10]. Several studies have demonstrated that recurrence risk after treatment is related to the depth of myometrial invasion, tumor type and tumor differentiation grade, lymphovascular space invasion (LVSI) as well as FIGO stage [16-19]. These prognostic factors allow the definition of recurrent risk groups and guide decision-making worldwide. 

Currently, the molecular factors that may influence the prognosis of the disease are under investigation. However, they are still not being used in most cancer centers worldwide [4].

The main criteria to determine high-risk patients are non-endometrioid histology, histopathological grade 3, LVSI, myometrial invasion ≥50%, and cervical stroma involvement [6-9]. Tumor histology and grade are determined with endometrial sampling [12]. As for the assessment of myometrial invasion and cervical stroma involvement, MRI can accurately help assess this information [20-22].

High-grade Endometrial Cancer

High-grade EC includes all non-endometrioid tumor histology (mucinous carcinoma, serous carcinoma, clear cell carcinoma, neuroendocrine tumors, mixed cell adenocarcinoma, and undifferentiated or dedifferentiated carcinoma) and histopathological grade 3 (poorly/undifferentiated carcinoma) [5,6].

Lymphovascular Space Invasion

The criterion of LVSI is purely anatomopathological. It may be advanced at the time of biopsy, however, if absent initially, this should be confirmed again at the time of the surgical staging specimen evaluation. Recent data report that LVSI is an independent predictor for local and distant recurrence [5,8,23].

Myometrial Invasion and Cervical Stroma Involvement 

**Figure 1:**
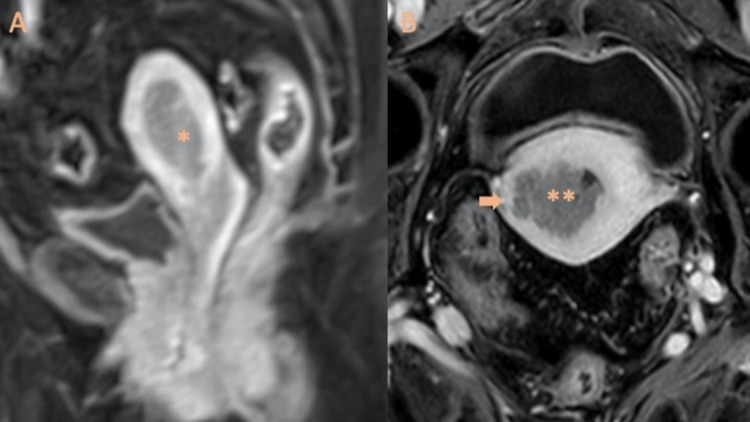
Stage I endometrial cancer A: Stage IA grade 2 endometrioid adenocarcinoma in a 77-year-old woman. Sagittal T1-weighted MRI with intravenous gadolinium-based contrast material demonstrates endometrial tumor (*) confined to the uterine cavity. B: Stage IB grade 3 endometrioid adenocarcinoma in an 82-year-old woman. The extent of myometrial invasion is well delineated after administration of intravenous contrast material. The endometrial carcinoma (**) infiltrates the outer one-half of the myometrium (arrow).

Radiology has a central role in determining the presence of these criteria during the pre-treatment evaluation, otherwise unknown until the time of surgery [24,25]. The depth of myometrial tumor invasion and the involvement of the cervical stroma are two parameters used for staging EC [25,26] and are important prognostic factors [12,25]. Myometrial invasion is measured from the endo-myometrial junction to the deepest point of invasion [25,27]. If the tumor is confined to the corpus uteri, or myometrial invasion is <50%, the tumor is assigned to stage IA. And if ≥50% has been invaded, it is categorized as IB (Figure 1) [9,28].

**Figure 2:**
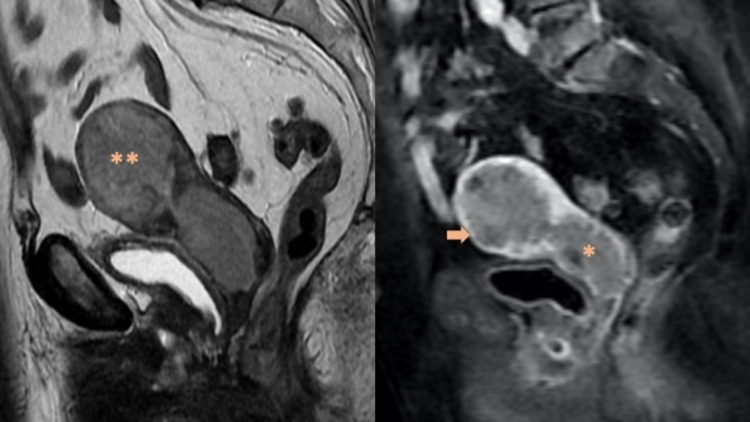
Stage II endometrioid adenocarcinoma in an 82-year-old woman. Sagittal T2-weighted MRI demonstrates intermediate to high-signal-intensity carcinoma (**) infiltrating the cervical stroma, which is better delineated after intravenous administration of contrast material (*). The tumor also invades the outer one-half of the myometrium (arrow).

Tumor grade and lymph node metastases correlate with the depth of myometrial invasion, which is the most important morphologic prognostic factor [11,28]. Therefore, the degree of myometrial invasion is directly correlated with the probability of advanced disease (in fact, the prevalence of lymph node metastasis is <2.5% in stage IA while it ranges from 15% to 45% in stage IB) [7,29,30]. If the tumor invades the cervical stroma but does not extend beyond the uterus, it is classified as stage II (Figure 2).

This stage doesn’t include tumors with endocervical glandular involvement, which are classified as stage I, as this has no prognostic impact. The evidence of pretreatment cervical invasion can lead to a different treatment plan and may further lead to preoperative radiation therapy [6].

Radiologic evaluation in high-risk patients

Pre-treatment

**Figure 3:**
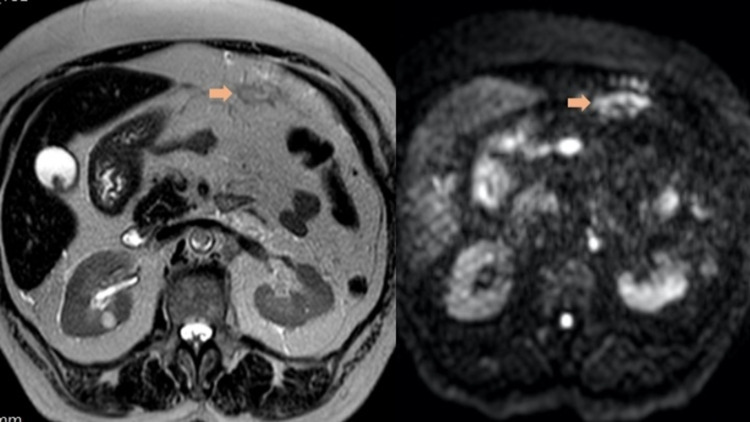
Endometrial serous carcinoma in a 67-year-old woman. Subtle peritoneal deposits (arrow) can be better delineated with diffusion-weighted imaging and appear as an area of restricted diffusion.

Regarding imaging techniques, transvaginal ultrasound remains the first imaging modality for the evaluation of patients with suspected EC. Nevertheless, MRI with intravenous contrast is the primary modality of choice for the anatomical assessment of the pelvic cavity and has an important role in identifying tumor infiltration into myometrium or cervical stroma and extension into parametria [12,25]. With a negative MRI and low-grade tumors, the risk of lymph node metastases is very low. Diffusion-weighted MRI (DW-MRI) is useful for detecting small metastatic implants in the omentum or lymph nodes, and also helps in evaluating myometrial infiltration as seen in Figure 3 [28].

Diffusion-weighted sequences are recommended by the European Society of Urogenital Radiology (ESUR) guidelines as part of the MRI protocol of EC diagnosis. The MRI protocol with thin-section high-resolution T2-weighted images, DW, and dynamic contrast-enhanced sequences improves staging patients with EC [13]. Furthermore, these preoperative images help plan surgery and postoperative adjuvant radiotherapy [12,15-17].

As per the National Comprehensive Cancer Network (NCCN) guidelines, MRI should be used in patients with type II EC or, if there’s a suspicion of cervical invasion [10-12]. 

On the other hand, ESUR guidelines also advise MRI in cases of type I EC to distinguish FIGO stage IA from IB since patients in the first case will not require lymphadenectomy. In addition, ESUR also recommends performing MRI in women of childbearing age with grade I EC who wish to preserve their uterus and fertility to help to exclude cervical and myometrial tumor infiltration. In these cases, if EC is confined to the uterine cavity, the possibility of fertility-sparing treatment must be included in the treatment options [13,28]. The American College of Radiology (ACR) also recommends MRI as the best modality approach for the initial staging of EC and treatment planning [7].

**Figure 4:**
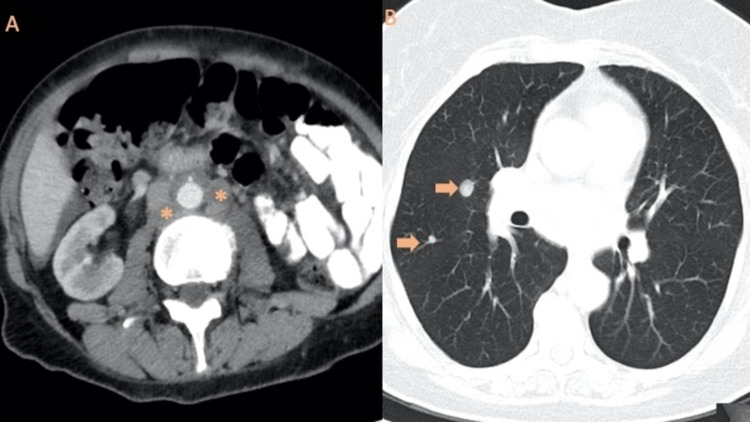
CT findings in two different patients with endometrial cancer A: CT with intravenous contrast in a 54-year-old woman with grade 2 endometrioid adenocarcinoma shows multiple para-aortic lymph nodes metastases (*) which corresponds to FIGO stage IIIC2. B: CT of the thorax in a 67-year-old woman with a clear cell carcinoma of the endometrium demonstrates disease distant spread with lung metastases (arrows).

Besides pelvic MRI to assess locoregional disease, contrast-enhanced CT of the abdomen and pelvis can be used to assess lymph node metastases in high-risk patients with a reported sensitivity and specificity of 30% to 57% and 92% to 98%, respectively [14,15,30]. The CT doesn’t have much accuracy to characterize an endometrial mass, as it is usually seen as a hypodense lesion or as an abnormally thickened endometrium frequently not distinguished from benign lesions. However, to visualize the entire pelvic and abdominal cavity to search for enlarged lymph nodes or other soft tissue masses, as well as lungs metastases, CT plays a vital role due to its good multiplanar spatial resolution (Figure 4) [12-15]. 

Regarding 18F-fluorodeoxyglucose (FDG) PET/CT, the overall pooled sensitivity and specificity for detection of lymph node metastasis were 72% and 94%, respectively [16,17]. Also, the maximum standardized uptake value (SUVmax) on (FDG) PET/CT is a predictor for overall survival [30-32] and has been correlated with a significantly lower disease-free survival rate. In fact, outcomes of patients with higher FDG uptake were significantly worse than those with lower uptake [17,18,31]. Kitajima et al. showed that patients with a high SUVmax (≥12.7) had a worse prognosis with higher relapse and mortality rates than those with a low SUVmax (<12.7; p=00.00042) [19]. The PET/CT has a good correlation with the clinical outcome of the patients and has a high negative predictive value (disease-free courses were reported more frequently in patients with negative PET/CT results) [20,33]. As per the revised ACR guidelines, both PET/CT and contrast-enhanced CT of the abdomen and pelvis are adequate imaging techniques for the preoperative evaluation of metastatic disease for a high-grade tumor in the staging of EC [7]. 

Follow-Up and Recurrent Endometrial Cancer

Approximately one-third of the patients with high-risk cancer may develop recurrent disease and radiology plays a pivotal role in the assessment of locoregional disease, distant metastasis, and the detection of persistence or recurrence of EC [21,22,30]. Endometrial cancer tends to recur in the pelvis, especially in the vaginal vault and pelvic lymph nodes, followed by para-aortic lymph nodes. Other common sites for extra-pelvic recurrence are the abdomen (especially peritoneum) and lungs [21-23,32]. Regarding surveillance of asymptomatic patients with treated high-risk EC, both radiography chest or chest CT with or without intravenous contrast may be appropriate to search for lung metastases. Besides, contrast-enhanced CT of the abdomen and pelvis can be obtained as a part of posttherapy surveillance in this subgroup of patients [7].

**Figure 5:**
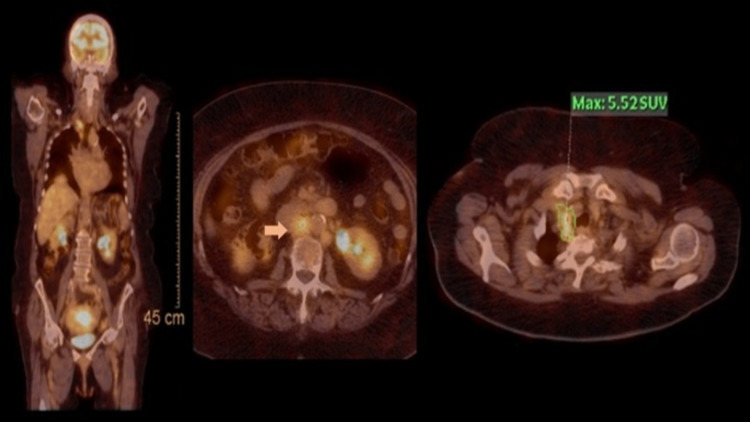
PET/CT from skull to mid-thigh performed in the context of clinical suspicion of EC recurrence Before beginning treatment, an endometrioid carcinoma G2 was diagnosed with the invasion of the outer one-half of the myometrium and vaginal metastasis in a patient. After chemo-radiotherapy, both clinical and radiological responses were obtained. One year post-treatment, the patient performed this PET/CT in the context of recurrence clinical suspicion. A hypermetabolic para-aortic lymph node (arrow) and a right supraclavicular lymph node with a SUVmax of 5.52 (image on extreme right) were detected, conferring the diagnosis of recurrence of the underlying disease.

Post-therapy evaluation in patients with a clinically suspected recurrence should include an abdominal and pelvic evaluation. In high-risk patients, it is appropriate to perform PET/CT from the skull base to the mid-thigh (Figure 5) in addition to the pelvic MRI [7]. 

In a recent meta-analysis, PET/CT has demonstrated a sensitivity and specificity of 95.8% and 92.5%, respectively, in detecting EC recurrence. Also, literature reports that FDG-PET implemented by CT or MRI has higher accuracy than CT or MRI alone in both evaluations of treatment response and detection of recurrence [23,32,33]. The MRI may have a role in the assessment of surgical resectability in the pelvis if it is only local tumoral recurrence. Chest CT with or without intravenous contrast is indicated in high-risk patients to detect lung metastases and is not necessary for low-risk groups as the incidence of lung metastases is very low in these patients [7]. Therefore, to confirm the clinical suspicion of known EC recurrence, CT of the chest, abdomen, and pelvis with intravenous contrast or, PET/CT as well as MRI of the pelvis or abdomen can be used in high-risk patients [7,20].

## Conclusions

Endometrial cancer is a frequent gynecological tumor and although considered to have an overall good prognosis, it is an increasingly significant cause of mortality for women. 

High-risk patients are a specific subgroup with a more aggressive clinical course as they are at a higher risk of disseminated disease and recurrence. It includes those with at least one defined radiological or pathological factor, including non-endometrioid histology, histopathological grade 3, lymphovascular space invasion, myometrial invasion ≥50%, and cervical stroma involvement.

Radiology plays a vital role in the assessment of these patients with MRI frequently used for evaluating locoregional disease, and CT or PET/CT for detecting lymph node involvement and distant metastases. Apart from preoperative staging, imaging is also useful for the detection of postoperative residual disease, detecting recurrent disease as well as in post-treatment surveillance of asymptomatic high-risk patients. These high-risk patients need to be managed by a multidisciplinary team to devise the best treatment approach. Also, radiology can help in patient stratification into treatment groups as well as improve staging accuracy.

In conclusion, radiologists should be aware of the recent advances in the diagnosis and management of endometrial carcinoma, especially in recognizing this specific higher-risk subgroup.
